# Bedaquiline does not enhance a clofazimine-azithromycin-ethambutol regimen against *Mycobacterium avium* in the hollow-fiber system

**DOI:** 10.1128/aac.01464-24

**Published:** 2025-04-14

**Authors:** J. Raaijmakers, S. Salillas, R. Aarnoutse, E. Svensson, L. Te Brake, R. Stemkens, H. Wertheim, W. Hoefsloot, J. van Ingen

**Affiliations:** 1 Radboudumc Community for Infectious Diseases, Department of Medical Microbiology, Radboud University Medical Center, Nijmegen, the Netherlands; 2 Department of Microbiology, Pediatrics, Radiology and Public Health, Faculty of Medicine, University of Zaragoza 189151, Zaragoza, Aragon, Spain; 3 Radboudumc Community for Infectious Diseases, Department of Pharmacy, Radboud University Medical Center, Nijmegen, the Netherlands; 4 Department of Pharmacy, Uppsala University 8097, Uppsala, Uppsala County, Sweden; 5 Radboudumc Community for Infectious Diseases, Department of Pulmonary Diseases, Radboud University Medical Center, Nijmegen, the Netherlands; St George's, University of London, London, United Kingdom

**Keywords:** PK/PD, *M. avium*, hollow-fiber system, nontuberculous mycobacteria, bedaquiline

## Abstract

Bedaquiline has been proposed as a second-line drug to treat pulmonary disease caused by *Mycobacterium avium* complex. Based on *in vitro* synergy and interactions, a logical regimen would combine bedaquiline and clofazimine as additions to an ethambutol-azithromycin backbone. Here, we evaluate the added benefit of bedaquiline in a regimen of azithromycin, ethambutol, and clofazimine. THP-1 cells infected with *M. avium* ATCC 700898 were seeded in a hollow-fiber model and exposed to a regimen of azithromycin, ethambutol, and clofazimine with or without bedaquiline for 3 weeks. Epithelial lining fluid pharmacokinetic profiles of azithromycin and ethambutol were simulated, while an average steady-state concentration was sought for clofazimine and bedaquiline. Pharmacokinetics and pharmacodynamics were monitored throughout the experiment. Both regimens led to sustained bacterial killing (both intracellular and extracellular) throughout the experiment. No difference in kill rate was observed between the two therapies. The extracellular kill rate for the 3-drug regimen was 0.65 (95% CI 0.63–0.67) and for the 4-drug regimen 0.65 (95% CI 0.64–0.67). The intracellular kill rate was 0.48 (95% CI 0.46–0.50) for the 3-drug regimen and 0.48 (95% CI 0.46–0.50) for the 4-drug regimen. Macrolide-tolerant subpopulations were observed with both treatment regimens at day 21. Bedaquiline does not add killing activity to a clofazimine-ethambutol-azithromycin regimen and did not improve suppression of the emergence of macrolide resistance, which makes its role as a second-line agent doubtful.

## INTRODUCTION

*Mycobacterium avium* complex (MAC) bacteria are the most frequent causative agents of nontuberculous mycobacterial pulmonary disease (NTM-PD) and are responsible for up to 85% of NTM-PD cases worldwide (1). The recommended treatment regimen is a combination of azithromycin (AZT), ethambutol (EMB), and rifampicin (RIF) with or without (inhaled) amikacin (2). Despite prolonged (>12 months) administration of this regimen, its overall outcome is poor (3). Within this treatment regimen, rifampicin shows limited activity, adverse effects, and pharmacokinetic interactions with macrolide antibiotics (4–7). Clofazimine (CFZ) has been proposed as a good alternative as it is active *in vitro*, shows synergy with macrolides and amikacin (8), and does not elicit negative pharmacokinetic interactions with azithromycin (6, 9). The replacement of rifampicin by clofazimine showed promising results in a hollow-fiber system of *M. avium* pulmonary disease (HFS-MAC), mouse models, and in patients (10–13).

A role for bedaquiline (BDQ), a potent antituberculosis drug, in MAC-PD treatment is the subject of ongoing debate. BDQ shows both *in vitro* and *in vivo* activity against reference and clinical strains (14–17), and promising outcomes in MAC patients have also been reported (18–20). It shows *in vitro* synergy with CFZ as both target the electron transport chain, which could be exploited to enhance clofazimine-based regimens (21, 22). Yet, if bedaquiline-containing regimens fail, this is often due to acquired resistance resulting from mutations in the TetR regulator of the mmpS5-mmpL5 drug efflux pump, which confers cross-resistance to clofazimine (23, 24).

Here, we used the HFS-MAC to determine the effect of adding BDQ to a CFZ-EMB-AZT regimen by evaluating antimycobacterial effect and suppression of macrolide resistance emergence of both regimens.

## MATERIALS AND METHODS

### Bacteria, cells, and antibiotics

The reference strain *Mycobacterium avium* subsp. *hominissuis* ATCC 700898 was purchased from the American Type Culture Collection (ATCC, Manassas, VA) and cultured in Middlebrook 7H9 supplemented with 10% OADC (Becton Dickinson, Vianen, the Netherlands) 5 days prior to the experiment. THP-1 cells were purchased from the German Collection of Microorganisms and Cell Cultures (DSMZ; Braunschweig, Germany; ACC 16 Lot 32) and cultured in RPMI 1640 supplemented with 20% heat-inactivated fetal bovine serum (FBS; Life Technologies Limited, Paisley, UK) at 36°C and 5% CO_2_.

AZT, EMB, and CFZ were purchased from Sigma-Aldrich (Zwijndrecht, the Netherlands). BDQ was provided by Janssen Therapeutics (Beerse, Belgium). Stock solutions of the compounds were prepared in ethanol, Milli-Q water, DMSO, and DMSO, respectively. Syringe solutions of AZT (40/60% [v/v] ethanol/Milli-Q water) and EMB (Milli-Q water) were prepared every 3 days. Due to drug instability, CFZ and BDQ bolus solutions at 100 and 10 µg/mL, respectively, were prepared daily in 10% DMSO, 0.5% Tween 80 in RPMI + 2% FBS.

### MIC determinations

The MICs of AZT, CFZ, and BDQ against *M. avium* ATCC 700898 were determined before the experiments by broth microdilution in cation-adjusted Mueller-Hinton broth according to CLSI guidelines (25). To assess the effect of FBS supplementation in RPMI 1640 on the MIC of BDQ and CFZ due to protein binding, MIC tests were conducted in RPMI 1640 containing 0%, 2%, or 10% FBS. Each condition was tested in duplicate for both CFZ and BDQ.

### Hollow-fiber setup

The hollow-fiber systems were set up and maintained as previously described (13). Briefly, THP-1 cells (used to represent human macrophages) at a density of 2 · 10^6^ cells/mL in RPMI + 10% heat-inactivated FBS were infected with a 0.5 McFarland suspension of *M. avium* and incubated for 24 hours at 36°C and 5% CO_2_ in a 1:3 ratio (bacteria:cells). Then, 4 hours prior to the start of the experiment, 30 mL of the infected THP-1 cells was introduced into each cellulosic C8008 hollow-fiber cartridge (FiberCell Systems, New Market, MD, USA).

AZT and EMB penetration into the extra-capillary space of the C8008 hollow-fiber cartridges (FiberCell Systems, New Market, MD, USA) after administering them to the central reservoir was confirmed prior to the experiment (Fig. S1 and S2). Every day, CFZ and BDQ were administered directly to the extra-capillary space as they cannot cross the hollow fibers, based on preparative experiments (see Supplement, paragraph 2). The cartridges were saturated with CFZ before simulating the pharmacokinetic profile to reduce the binding of CFZ and BDQ to plastics (13).

### Hollow-fiber study design and simulated pharmacokinetic profiles

A growth control arm and two experimental arms were included; a 3-drug regimen (AZT, EMB, and CFZ) and a 4-drug regimen where bedaquiline is added (AZT, EMB, CFZ, and BDQ). Both experimental regimens and the growth controls were performed in triplicate.

Protein-unbound epithelial lining fluid (ELF) drug concentrations of AZT (250 mg/day) and EMB (15 mg/kg/day) were mimicked, taking a 25% increase in AZT exposure into account when not administered with rifampicin (6, 26–29). The pharmacokinetic profiles were simulated as described earlier (Table 1) (13). The inflow of fresh growth medium was set to the half-life of ethambutol, and the difference in half-life between EMB and AZT was corrected for by zero-order top-up.

**TABLE 1:** Values represent geometric mean ± standard deviation^*a*^

Parameter	Drug	Target	3-Drug regimen	4-Drug regimen
			Actual	Actual
T_1/2_ (h)	Azithromycin	20^(38)^	22.8 ± 2.4	18.5 ± 3.0
	Ethambutol	10^(39)^	13.0 ± 0.7	12.1 ± 1.0
T_max_ (h)	Azithromycin	10^(38)^	9.2 ± 2.3	10.6 ± 1.2
	Ethambutol	3^(39)^	3 ± 0	3.3 ± 0.6
C_max,ss_ (mg/L)	Azithromycin	3.75^(38)^	4.1 ± 0.2	4.2 ± 0.7
	Ethambutol	3^(39)^	3.4 ± 0.3	3.3 ± 0.2
AUC_0-24_ (mg·h/L)	Azithromycin	62.4	75.2 ± 2.7	73.4 ± 4.6
	Ethambutol	48.5	47.2 ± 2.7	46.5 ± 3.0
C_avg_ (mg/L)	Clofazimine	2.2	2.74	2.49
	Bedaquiline	0.06	^*b*^-	0.102

^*a*^ T_1/2_, elimination half-life; T_max_, time at which C_ss_ is reached; C_ss_, peak concentration at steady state; AUC_0-24_, area under the 24 hour-concentration-time curve at steady state; C_avg_, average concentration at steady state.

^*b*^ -, no value.

An average concentration at protein-unbound clinical steady state (C_avg_) was targeted for CFZ (100 mg/day) and BDQ (200 mg t.i.w.). The pharmacokinetic profile targeted for CFZ has been described before (13). Similar extrapolation steps have been applied to define a BDQ target as plasma concentrations were extrapolated to protein-unbound lung concentrations. A plasma C_avg_ of 0.9 mg/L has been considered as this is the average concentration reached at steady state in approved regimens (30–36). Furthermore, we took into consideration a lung/plasma AUC_0-168h_ of 34, which has been obtained in the murine model after repeated doses of BDQ at 25 mg/kg/day and is in line with the information described by Rouan et al. (37). To take drug binding into consideration, we applied a binding of 99.9%, based on observed plasma protein binding (35, 38). Since albumin is the main protein in plasma and BDQ strongly binds to albumin (36), we took a 0.6 ratio into account that there is more protein-unbound BDQ in ELF, based on the ratio between plasma albumin and ^125^I-albumin concentration in ELF (39). BDQ has proven to bind to plastic and glass, which was compensated for by increasing the dose by 20% (Fig. S5). This results in a C_avg_ of 0.0612 mg/L. Preparative experiments showed BDQ instability in the HFS following first-order kinetics with a T_1/2_ of 24.8 hours (Fig. S4). Therefore, a BDQ concentration of 0.084 mg/L was targeted to achieve the desired C_avg_ during the dosing interval. For both CFZ and BDQ, a bolus was administered every 24 hours to achieve an average concentration at clinical steady-state.

### Bacterial and THP-1 cell enumerations

Bacterial densities and THP-1 cell counts were determined at days 0, 3, 7, 14, and 21 as described earlier (26), and a brief overview is presented in the supplementary materials. Using the R package NLMIXR2, the net growth rate (K_net_) and the maximum bacterial carrying capacity (B_max_) of the intracellular and extracellular fractions were estimated by fitting a growth model to the raw data according to formula (F1). Subsequently, the treatment effects were estimated by fixing the K_net_ and B_max_ values from the first model and subtracting antibiotic effect from the fixed growth rate (F2).


(F1) $$\frac{d}{dt}B=K_{net}∙\left(1-\frac{B}{B_{max}}\right)∙B$$

(F2) $$\frac{d}{dt}B={(K}_{net,fix}-Antibioticeffect)∙\left(1-\frac{B}{B_{max,fix}}\right)∙B$$

The emergence of macrolide tolerance was determined by inoculating the bacterial samples onto Middlebrook 7H10 agar (M7H10; Becton Dickinson, Vianen, the Netherlands) plates containing AZT at a final concentration of eight times the initial MIC.

### Pharmacokinetic measurements

Drug concentrations of AZT, EMB, CFZ, and BDQ were determined at day 0 of the experiment and at steady state (day 21) by ultra-high performance liquid chromatography-mass spectrometry as previously described (supplemental paragraph 5.2.4). To determine BDQ concentrations, samples were taken at 15 minutes, 12 hours, and 24 hours after bolus injection from the extra capillary space of the cartridges (Fig. S6). The analysis of all drugs is described in the Supplement, paragraph 5.2.4.

### Calculations and statistics

Plotting of pharmacodynamic, THP-1 cell density, and pharmacokinetic data was done using GraphPad Prism version 7.0.0 (GraphPad Software Inc., La Jolla, CA, USA). Non-compartmental analyses were performed using Phoenix 64 WinNonlin (Build 8.3.1.5.014) as described earlier (13). CFZ and BDQ C_avg_ were calculated by dividing their AUC_0-24_ by 24 hours using GraphPad Prism version 7.0.0 (GraphPad Software Inc., La Jolla, CA, USA). All pharmacokinetic parameters were depicted as the geometric mean and a standard error of the mean.

## RESULTS

### MIC determinations

The MICs of AZT, EMB, CFZ, and BDQ were 64, 8, 0.12, and 0.06 mg/L, respectively. The MICs of CFZ and BDQ in RPMI 1640 supplemented with 0%, 2%, and 10% FBS were 0.25, 0.25, and 0.5 for CFZ, respectively, and 0.12, 0.12, and 0.25 for BDQ, respectively.

### Pharmacodynamic effect and emergence of macrolide tolerance

**Fig 1:**
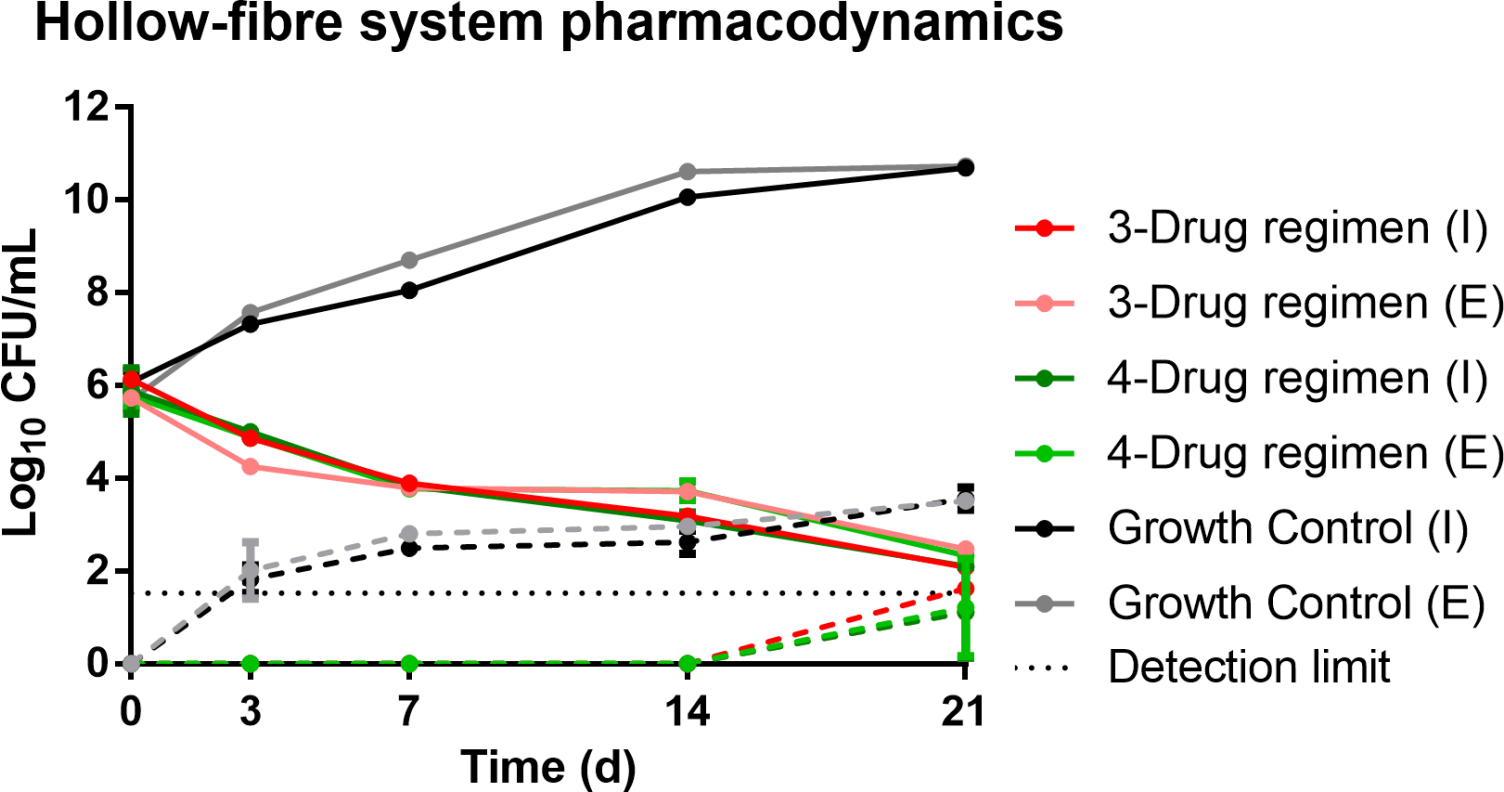
Hollow-fiber pharmacodynamic measurements (solid lines) and emergence of macrolide resistance (dashed lines) of the growth control, clofazimine-based therapy, and bedaquiline added therapy in the hollow-fiber experiment. I, intracellular Log_10_CFU/mL; E, extracellular Log_10_CFU/mL counts.

Both treatment regimens were able to reduce the total intracellular and extracellular bacterial concentrations over the course of the experiment (Fig. 1). The kill rate for the intracellular bacteria was 0.48 (95% CI 0.46–0.50) for the clofazimine treatment and 0.48 (95% CI 0.46–0.50) for the bedaquiline treatment. The extracellular kill rate was 0.65 (95% CI 0.63–0.67) for clofazimine treatment and 0.65 (95% CI 0.64–0.67) for bedaquiline treatment. No statistical differences in kill rates were observed. A small proportion of the population showed tolerance to azithromycin on day 21.

### Pharmacokinetic evaluation

The pharmacokinetic targets were attained for azithromycin, ethambutol, and clofazimine in both trial arms. The achieved bedaquiline exposure was 70% higher than the target. The complete pharmacokinetic curves can be found in the supplementary files (Fig. S7).

### THP-1 cell densities

THP-1 cell densities remained stable throughout the experiment. Every other day, all cartridges except the growth controls were replenished with THP-1 cells to compensate for THP-1 cell death induced by the addition of DMSO to the cartridges and the vigorous mixing required upon administration of a clofazimine and bedaquiline bolus. The densities of THP-1 cells are shown in Fig. S6.

## DISCUSSION

Bedaquiline does not add any activity to a clofazimine-ethambutol-azithromycin regimen for MAC-PD in the HFS. The lack of an additive effect applies both to the killing rates and to the suppression of macrolide resistance in the course of the experiment.

The exposures of azithromycin, ethambutol, and clofazimine met preset targets and were similar for both arms. However, the bedaquiline exposure was 70% higher than targeted. This overexposure has no bearing on the conclusion, as even higher doses of bedaquiline as targeted had no effect on the kill rate of the baseline regime. The lack of effect, even at bedaquiline concentrations twice its MIC, could be attributed to altered bacterial drug susceptibility within the intracellular environment or an incomplete drug equilibrium between the extracellular and intracellular compartments of the THP-1 cells (40). This lack of additive effect contrasts with *in vitro* studies that found low bedaquiline MICs and synergy with clofazimine (21, 22) as well as the preliminary clinical observation that bedaquiline might strengthen regimens in select patients in a cohort (20). Of note, a follow-up study of the initial clinical cohort revealed frequent treatment failure with acquired mutational resistance to BDQ (23); similar observations were done in other published cases (24). Apparently, despite the very low MICs observed for bedaquiline, pharmacodynamic targets were not achieved despite the high exposures in our experiment. This study highlights the need to evaluate the effect of antibiotics in *in vivo*-like situations such as intracellular environments and the effects within the recommended treatment antibiotics. We chose to add to a clofazimine rather than rifampicin-based regimen because of its superior efficacy in prior HFS studies, clofazimine-bedaquiline synergy *in vitro*, encouraging outcomes in a recent clinical trial, and the known negative pharmacokinetic interactions between rifampicin and bedaquiline, as rifampicin strongly decreases bedaquiline exposure (41).

Brown-Elliot et al. and Litvinov et al. demonstrated that bedaquiline exhibits low MICs against clinical MAC isolates (MIC_90_s of 0.015 and 0.12 mg/L, respectively), which are comparable to the MIC observed for the reference strain used in our experiment (0.06 mg/L) (42, 43). Bedaquiline is mentioned in international guidelines for MAC-PD as a possible second-line drug to be used for patients intolerant to, or with strains resistant to, first-line drugs (2). Our *in vitro* findings do not support this notion, at least not for the modelled exposures, based on pharmacokinetic data of the recommended dosing for tuberculosis treatment. Higher dosing or increased exposure by more direct application of bedaquiline (e.g., inhalation) to the site of infection might still be effective, but its safety has never been established (44). Still, the clinical observation in case reports of the occurrence of BDQ resistance after adding to a failing multidrug regimen is of great concern, which in combination with the results from this study suggest that BDQ is not suitable for treatment of NTM-PD disease.

This study has limitations. All limitations (e.g., limited amount of strain variation) that apply to *in vitro* models with a controlled environment also apply to the current model. The steady-state (protein-unbound) concentrations of azithromycin and ethambutol were simulated based on pharmacokinetic studies in ELF, whereas such studies and data were not available for clofazimine and bedaquiline. To our knowledge, no ELF pharmacokinetics are known for these drugs, and extrapolations from various sources in the literature were used, which may not be identical to the human *in vivo* situation at the site of infection. Another limitation is that we did not test for the emergence of bedaquiline resistance and cross-resistance between clofazimine and bedaquiline (23). If bedaquiline induces cross-resistance, its addition to therapy might not enhance efficacy but instead diminish it by triggering cross-resistance to clofazimine. Consequently, the therapeutic effect of clofazimine, an already proven effective drug, could be compromised (13, 41). However, there is no standardized method for bedaquiline MIC testing, and broth microdilution tests of bedaquiline are sensitive to variations in pH and the types of plastics used. Consequently, changes in MIC values need not accurately reflect shifts in bedaquiline susceptibility but rather highlight the limitations of MIC testing methods. Molecular characterization could have provided more valuable insights, even though genotype-phenotype correlations are incompletely understood (23).

To conclude, bedaquiline does not enhance the antibacterial effect of a regimen of azithromycin, ethambutol, and clofazimine against MAC in a hollow-fiber model of pulmonary disease. These results do not support the initial enthusiasm for bedaquiline based on the case series (20) and do not support its current position as a second-line drug in international guidelines. The evaluation of bedaquiline in the current dose and route of administration for the treatment of MAC-PD should be low on the priority list for clinical trials. Above all, clinicians should be hesitant to add BDQ to a failing treatment regimen.

## SUPPLEMENTAL MATERIAL

Supplemental material aac.01464-24-s0001.docx Fig. S1 to S10; Tables S1 to S22.

ASM does not own the copyrights to Supplemental Material that may be linked to, or accessed through, an article. The authors have granted ASM a non-exclusive, world-wide license to publish the Supplemental Material files. Please contact the corresponding author directly for reuse.
